# On the Treatment of Itch

**Published:** 1851-06

**Authors:** 


*On the Treatment of Itch.* By MM. Bazin and Bourguignon.—M. Bazin, physician to the St. Louis, in a recent report, furnishes an account of the trials he has made of the various means of treating the itch, and of the definitive results he has arrived at. He states that, at the time of his appointment in 1847, the medium time occupied in treating the disease by the *sulphuro-alkaline ointment* applied to the wrists and insteps, together with sulphureous baths and fumigations, was fourteen days; but that since he has caused the *entire body* to be well rubbed with it (rubbing with extra force those parts the acari specially infest), the patients are dismissed cured in two or three eays. Some practitioners who have adopted the plan have erred in its application by leaving some portions of the body unrubbed, or by continuing the friction as long as any itching was perceived. In the first case the disease reappears; while in the other, by prolonging the frictions beyond the time necessary to destroy the acari, and the vitality of their ova, other eruptions are induced which give rise to great itching. This itching and eruption, occurring after the employment of two complete frictions, furnish an indication to desist instead of to continue; and if they do not then disappear, they may be relieved by tepid baths.

There are cases, however, in which, from the existence of great abundance of pustules, the sulphuro-alkaline ointment would excite too much pain, or in which the patients have such an invincible repugnance to its smell, that we should resort to some other substance; and M.

Bazin finds, by numerous trials, that the *lard* and *oil*, which form the base of all antipsoric preparations, if employed in general frictions, either together or separate, will effect a cure, only from four to six, instead of two, frictions being required. Another ointment recently tried, containing *chamomile*, cures in three frictions, soothes the itching instantly, and does not give rise to any secondary eruption. It is composed of equal parts of fresh chamomile, olive oil, and lard.

M. Bourguignon, in his recent prize essay, prefers the *staphisagria* to any other remedy. He adds 300 parts to 500 of lard, stirring the powder into the boiling lard, and keeping up a temperature of 100° C. for twenty-four hours. After straining, a little essence may be added. Baths should be taken before and during the treatment, and the frictions should be made four times a day, the cure being completed by the fourth day.—*British and Foreign Med. Chirurg. Rev.*, Jan. 1851, from *Am. Journ. Med. Sci.*

